# Evaluation des résultats de l'implantation cochléaire

**DOI:** 10.11604/pamj.2015.22.110.8029

**Published:** 2015-10-08

**Authors:** Khaoula Hssaine, Btissam Belhoucha, Othman Benhommad, Youssef Rochdi, Hassan Nouri, Lahcen Aderdour, Abdelaziz Raji

**Affiliations:** 1 Service d'ORL et de Chirurgie Cervico-faciale, CHU Mohammed VI de Marrakech, Maroc

**Keywords:** Implantation cochléaire, surdité, rééducation orthophonique, langage, cochlear implant, deafness, speech therapy, language

## Abstract

Les implants cochléaires sont des prothèses électro-acoustiques qui ont pour rôle de pallier une déficience bilatérale de l'oreille interne, qu'elle soit profonde ou sévère, acquise ou congénitale. Nous rapportons l'expérience du service d'Oto-rhino-laryngologie et de chirurgie cervico-faciale de l'hôpital universitaire Mohammed VI de Marrakech, dont l'objectif est d'évaluer l'implantation cochléaire et de préciser les facteurs influençant les résultats dans notre pratique. Le profil APCEI a été utilisé pour évaluer les résultats orthophoniques. Il s'agit d'une étude rétrospective étalée sur une période de 7 ans (Décembre 2007 à Décembre 2014). Durant cette période; 54 patients ont été implantés et suivient dans notre formation. Il s'agit de 30 filles et 24 garçons atteints d'une surdité sévères à profonde bilatérale, dont 48 enfants avaient une surdité pré-linguale. L'âge moyen d'implantation cochléaire pédiatrique était de 5,15 ans. L'implantation était unilatérale chez tous les patients. L'intervention était suivie par des réglages et une rééducation orthophonique régulière. L'évaluation était réalisée par la même équipe chaque mois durant les premiers 6 mois, puis tous les 6 mois. La durée moyenne de suivi était de 30,86 mois. Tous les patients ont tiré un bénéfice de leurs implants avec une variabilité interindividuelle. Les bons résultats étaient corrélés à l'implantation précoce, à un investissement parental important et au un bon suivi de la rééducation orthophonique. L'implantation cochléaire a révolutionnée la prise en charge de la surdité profonde et sévère. C'est une technique sûre, efficace lorsqu'elle s'adresse à des populations correctement sélectionnées.

## Introduction

Les implants cochléaires sont des prothèses électro-acoustiques qui permettent de pallier une déficience bilatérale de l'oreille interne, qu'elle soit profonde ou sévère, acquise ou congénitale. Ils transforment les ondes sonores en signaux électriques qui stimulent directement le nerf auditif. L'implantation cochléaire a maintenant démontré son efficacité dans la réhabilitation de ces surdités chez l'adulte et l'enfant. C'est une technique sûre, efficace lorsqu'elle s'adresse à des populations correctement sélectionnées. Elle nécessite l'intervention d'une équipe multidisciplinaire disposant d'un plateau technique et audio phonologique adapté. Le but de notre étude est d'évaluer les résultats orthophoniques en post-implantation cochléaire dans notre pratique.

## Méthodes

Il s'agit d'une étude rétrospective réalisée au centre hospitalier universitaire Mohammed VI de Marrakech. Cette étude s'est étalée sur une période de 7 ans allant de décembre 2007 à décembre 2014. Le recueil des données a été réalisé à partir des dossiers d'hospitalisations des patients, et des fiches d'évaluation orthophoniques remplis au cours des contrôles des patients en présence des chirurgiens, des orthophonistes et des parents des enfants. Les patients ont été convoqués régulièrement pour contrôle à 1 mois, 3 mois puis tous les 6 mois en post-implantation cochléaire. Le profil APCEI a été utilisé pour évaluer les résultats orthophoniques. Il aborde cinq domaines: Acceptation de l'appareil de l'implant, Perceptions auditives, Compréhension du message oral perçu (sans lecture labiale), Expression orale, et Intelligibilité de la parole. Chacun de ces domaines va être coté entre 0 et 5, 0 correspondant à l'absence de performance et 5 correspondant à la performance maximale. La durée moyenne de suivi était de 30,86 mois (7 ans-1 mois).

## Résultats

### Données épidémiologiques

Durant cette période, 54 patients ont été implantés au service d'ORL et de chirurgie cervico-faciale au sein du CHU Mohammed VI de Marrakech. Il s'agit de 30 filles et 24 garçons atteints d'une surdité profonde bilatérale, dont 48 enfants ayant une surdité pré-linguale. L'âge moyen d'implantation cochléaire pédiatrique était de 5,15 ans (1an 6mois-13ans), avec un adulte implanté à l'âge de 19 ans. Les étiologies de la surdité étaient: anoxie néonatale chez un cas, syndrome de Waadenberg chez 3 cas, rubéole congénitale chez deux cas, syndrome de Susac chez un cas, surdité post-méningite bactérienne chez deux cas, et l'origine était indéterminée chez le reste des patients. Le bilan génétique n'a pas était réalisé par faute de moyens.

### Bilan de pré-implantation cochléaire

Tous les patients ont bénéficié d'un bilan en pré-implantation cochléaire (examen clinique, audiologique, radiologique, évaluation orthophonique et psychologique). Le bilan n'a pas objectivé de contre indication pour l'implantation cochléaire. L'essai prothétique était réalisé chez 6 patients. Les comorbidités associées chez les patients étaient un cas de cécité unilatérale, un cas de cardiopathie et un cas de parésie de l'hémicorps gauche. Une malformation cochléaire complexe unilatérale était identifiée à l'imagerie chez une patiente implantée du coté contre latéral.

### Chirurgie de l'implant cochléaire

Cinq patients on été traités pour une otite séro-muqueuse associée en pré-implantation cochléaire. La vaccination anti-pneumococcique était systématique. L'implantation était unilatérale chez tous les patients, droite chez 48 cas et gauche chez 6 cas. Trois cas d'explantations et de réimplantations du même coté pour une panne du matériel implanté, dont une patiente implantée dans notre formation. La chirurgie s'est déroulée sous anesthésie générale avec monitoring du nerf facial et sans incidents. La voie d'abord était la voie rétro-auriculaire avec une tympanotomie postérieure pour exposer la fenêtre ronde. L'insertion des portes électrodes était complète dans la rampe tympanique chez tous les patients sauf deux électrodes pour un cas de réimplantation cochléaire et un cas d'implantation en post-méningite bactérienne.

### Suivi en post-implantation cochléaire

La durée moyenne d'hospitalisation était de 48 heures. La radiographie standard de Sternvers était réalisée systématiquement qui objectivait le bon emplacement de l'implant cochléaire chez tous les patients. Aucune complication en post-implantation n'a était constatée durant le suivi de nos patients. Le premier réglage ou activation de l'implant cochléaire était réalisé après un mois de la chirurgie avec une rééducation orthophonique régulière de à raison de 2 séances par semaine en moyenne. Les réglages étaient réguliers et modifiés par la suite selon l'évolution en rééducation orthophonique, schématiquement à 1, 2, 3, 6, 9, 12, 18, 24 mois puis annuellement.

### Evaluation des résultats orthophoniques en post-implantation cochléaire

**Figure 1:**
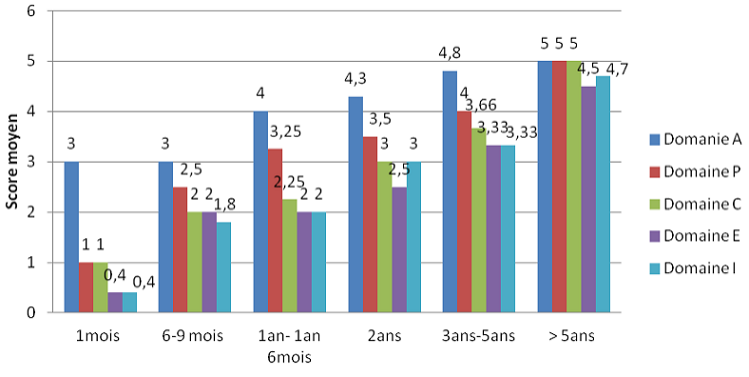
Score moyen APCEI des patient implantés en fonction de la durée du port de l'implant cochléaire

**Figure 2:**
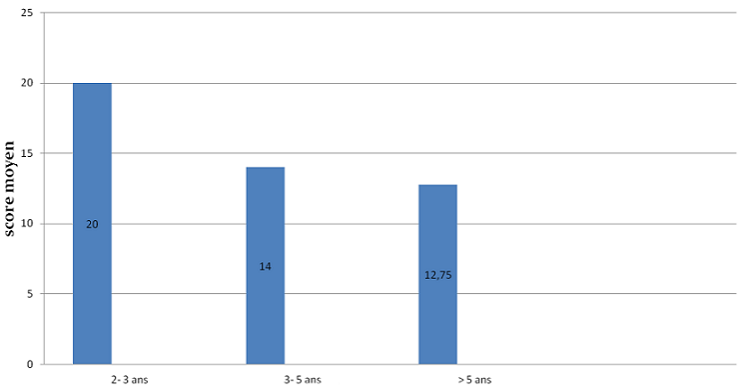
Score moyen APCEI en fonction de l’âge d'implantation

**Figure 3:**
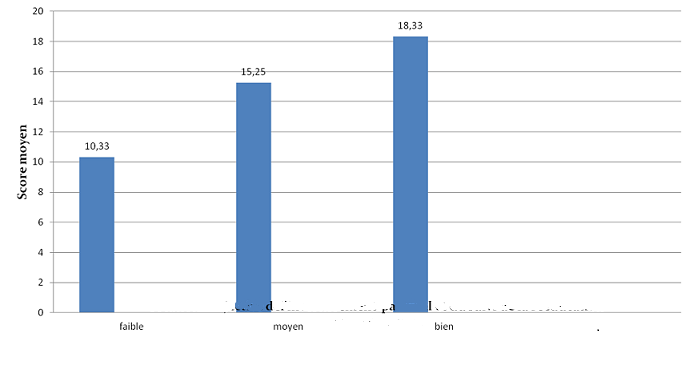
Score moyen APCEI par rapport à l'investissement parental et le suivi de la réeducation orthophonique

**Figure 4:**
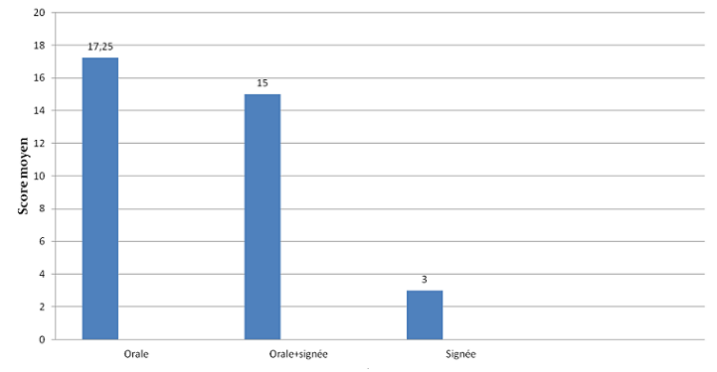
Score moyen APCEI en fonction du mode de la communication

L'évaluation orthophonique par le questionnaire APCEI a été réalisée en pré-implantation cochléaire et au cours du suivi. Tous les patients avaient un score à 0 avant l'implantation cochléaire dans tous les domaines du questionnaire utilisé. L'évaluation était réalisée chaque mois durant les premiers 6 mois, et par la suite tous les 6 mois. La Figure 1 illustre l'évolution du score APCEI au cours du dernier contrôle des patients et en fonction de la durée du port de l'implant cochléaire. Tous les patients de notre expérience ont tiré un bénéfice de leurs implants avec un recul moyen de suivi de 30,86 mois. Les résultats orthophoniques s'amélioraient avec la période d'utilisation de l'implant cochléaire. Les résultats présentaient une grande variabilité interindividuelle (âge d'implantation, durée du port de l'implant, motivation, rythme de la rééducation orthophonique. Les variables qui nous paraissaient les plus pertinents ont été étudiés chez nos patients on comparant les résultats de populations homogènes. La Figure 2 représente le score moyen APCEI de la somme des différents domaines chez enfants sourd prélinguaux en fonction de l'âge d'implantation. On constate que les bons résultats étaient corrélés à l'implantation précoce et donc à une courte durée de privation sonore. Les enfants ayant un investissement parental important et un bon suivi de la rééducation orthophonique ont eu les meilleurs résultats par rapport aux enfants ayant un suivi faible ou modéré (Figure 3). L'environnement oral a eu une influence positive sur les résultats de l'implant contrairement à la communication signée (Figure 4). Concernant nos résultats globaux, tous les patients acceptaient le port de l'implant cochléaire avec une acquisition de l'alerte à l'environnement sonore dès les premier mois d'utilisation de l'implant cochléaire. La compréhension de mots a été débutée à partir de 1 an d'utilisation, et la possibilité de comprendre une conversation à partir de la 3éme année. L'expression orale était constatée de manière décalée par rapport à la perception, et l'intelligibilité de la parole produite est corrélée aux performances perceptives obtenues. L'implantation cochléaire a permis une scolarisation en milieu ordinaire chez 80, 95% des implantés. Le taux d'échec était de 1,85%.

## Discussion

Inclure votre discussion ici La surdité est le déficit sensoriel le plus fréquent, sa prévalence est de 1/1000 naissances et de 5/1000 adultes. Les surdités de perception sévères et profondes sont responsables d'un handicap social et psychologique majeur surtout chez les enfants avant le développement du langage. La réhabilitation de ces surdités par l'implant cochléaire permet de court-circuiter les cellules ciliées endommagées ou absentes pour stimuler directement le nerf auditif. Les indications de l'implantation cochléaire figurent dans le rapport de la Haute Autorité de Santé (HAS), il s'agit des cas des surdités profondes (déficit bilatéral moyen de plus de 90 décibels) et des surdités sévères (déficit auditif moyen entre 70 de 90 décibels) avec une discrimination inférieure ou égale à 50% lors de la réalisation de tests d'audiométrie vocale adaptée à l'âge du patient. L'âge d'implantation cochléaire doit être le plus précoce possible en cas de surdité pré-linguales (avant 2 ans), et il n'y a pas de limite d'âge en cas de surdité post-linguale (après 5 ans) et chez l'adulte [1, 2] La sélection des patients potentiellement implantables et leur information, la chirurgie, le réglage leur rééducation, et leur évaluation se fera par la même équipe et au sein du même centre. L'équipe multidisciplinaire est composée de chirurgiens, anesthésistes, radiologues, orthophonistes, électrophysiologistes, psychologues, audioprothésistes et équipe paramédicale. Cette équipe se réunit régulièrement afin de coordonner son action et de permettre un suivi personnalisé des patients dans des structures appelées «centres d'implantation cochléaires». Le bilan de pré-implantation cochléaire comprend un bilan clinique complet, un bilan audiologique confirmant la surdité avec recherche d'une pathologie associée de l'oreille moyenne, un bilan radiologique comprenant une TDM des rochers et une IRM encéphalique à la recherche d'une malformation ou des calcifications de la cochlée, une aplasie de la cochlée ou du nerf auditif contre indiquant l'implantation, une évaluation psychologique pour éliminer des troubles majeurs pouvant contre indiquer la chirurgie, une évaluation de l'investissement du malade et des parents pour les enfants sourds, et une évaluation orthophonique pour préciser le mode de communication et le niveau de perception et de production orale, ce qui permet une comparaison des résultats orthophoniques en post implantation [3, 4]. L'acte chirurgical permet la mise en place de la partie interne de l'implant cochléaire, dont le but est de fixer le récepteur dans la région rétro-mastoïdienne avec une tympanotomie postérieure qui permet d'exposer la fenêtre ronde, et d'introduire le porte-électrodes dans la rampe tympanique pour reproduire la tonotopie fréquentielle naturelle de l'oreille [2]. La méningite est la principale complication post-opératoire, devenant rare avec la vaccination prophylactique systématique, le colmatage de la cochléostomie et le respect des règles d'asepsie [5]. Le suivi des patients implantés après la chirurgie constitue la clé de la réussite de toute implantation cochléaire. L'activation des électrodes est débutée dès cicatrisation de la plaie, ensuite les réglages sont fréquents dans les mois qui suivent l'implantation puis s'espacent selon l'évolution de la perception du patient. La rééducation orthophonique doit être régulière et à long terme surtout chez l'enfant. Les protocoles d'évaluation en post-implantation cochléaire sont multiples [6–8], variant d'un centre et d'un pays à l'autre (TEEP, MUSS, APCEI, CAP, MAIS, SIR..). Il n'existe en particulier pas de protocole standardisé ni adapté à notre culture. Le profil APCEI a été choisi dans notre évaluation malgré qu'il soit subjectif mais il reste un test simple, rapide à réaliser et explore les différents domaines d'évaluation orthophonique (perceptions auditives, compréhension du message oral perçu, expression orale, et Intelligibilité de la parole). L'implantation cochléaire est une technique sûre, efficace lorsqu'elle s'adresse à des populations correctement sélectionnées, son efficacité a été déjà démontrée et nos résultats concordent avec celles de la littérature. Globalement l'implant cochléaire permet l'amélioration du langage et de l'intelligibilité de la parole, avec accès à une scolarisation en milieu normal chez les enfants [9, 10], et l'augmentation des opportunités d'emploi, et une moindre dépendance des services sociaux à l'âge adulte [11]. Le taux d'échec ou de non utilisation est trop faible (1% à 2,7% par an dans le monde). Notre faible échantillon ne permet pas de sortit avec des conclusions ni d'étudier tous les facteurs influençant les résultats en post-implantation cochléaire. Les données de la littérature confirment que les bons résultats étaient corrélés essentiellement à l'implantation précoce, à un investissement parental important et au un bon suivi de la rééducation orthophonique et des réglages. La connaissance de tous les facteurs (âge d'implantation, durée de privation sonore, rééducation orthophonique, motivation, comorbidité [7, 8]..) permet d'adapter le suivi des patients cas par cas pour améliorer encore les résultats de l'implantation cochléaire.

## Conclusion

L'implantation cochléaire a maintenant démontré son efficacité dans la réhabilitation des surdités sévères et profondes chez l'adulte et l'enfant. Elle nécessite l'intervention d'une équipe multidisciplinaire disposant de moyens matériels et humains adaptés aux besoins. Des normes internationales sont fixées sur la composition des équipes multidisciplinaires expérimentées dans le dépistage, l'évaluation, la chirurgie et le suivi à long terme de l'implanté cochléaire, ainsi sur l'environnement technique obligatoire et l'activité en nombre minimum d'actes chirurgicaux. Le développement des programmes de dépistage de la surdité en milieu néonatal et la création de centres d'implantation cochléaires aux normes internationaux, devrait aider grandement à encore améliorer les résultats globaux de l'implantation cochléaire.
